# Sampling the skin surface chemistry for diagnosis and prognosis

**DOI:** 10.1111/wrr.13030

**Published:** 2022-06-17

**Authors:** Guy H. M. Stanley, Katie Wang, Patrick Daly, Christopher Lau, Aoife M. O'Brien, Cheryl Hamill, Mark Fear, Fiona M. Wood

**Affiliations:** ^1^ State Adult Burns Unit Fiona Stanley Hospital, SMHS Western Australia Australia; ^2^ Burns Injury Research Unit University of Western Australia Crawley Western Australia Australia; ^3^ Royal Perth Hospital East Metropolitan Health Service Western Australia Australia; ^4^ Department of Plastic & Reconstructive Surgery Fiona Stanley Hospital, SMHS Western Australia Australia; ^5^ Library & Information Service SMHS Western Australia Australia

**Keywords:** biomarkers, nitrocellulose membrane, skin blotting, surface chemistry, wound blotting

## Abstract

Skin and wound blotting are non‐invasive techniques used to sample the skin and wound surface chemistry, whereby a nitrocellulose membrane is applied to an intact or broken cutaneous surface to detect biomarkers. However, there has been no comprehensive review of the evidence for the techniques used and data obtained to date. The primary aim of this study was to review the utilities of surface blotting for the diagnosis and prognosis of physiological, pre‐disease, and pathological states. The secondary aim was to summarise the procedural steps. A systematic literature search was conducted on 9 July 2021 using Medline, Embase, and Google Scholar databases. Investigators used McMaster's Critical Review Form for Quantitative Studies to assess quality, then performed a narrative synthesis reporting according to Preferred Reporting Items for Systematic Reviews and Meta‐Analyses (PRISMA) guidelines. Twenty‐five studies were reviewed. Eighteen studies were of good quality, and seven were of moderate quality. These studies conducted skin and wound blotting on 176 animals and 1546 humans. Studies reported physiological and pathological states for diagnosis and prediction of conditions, including skin tears, wound healing, biofilm detection, and skin barrier function. The four steps for blotting are surface preparation, blot preparation, application and removal of blot, and analysis. This review demonstrates that blotting can determine the skin and wound surface chemistry using a versatile and reproducible technique. However, future research is needed to validate the technique and skin biomarkers identified.

**List of abbreviations**
CK‐M: creatine kinase M type
COL‐4: type IV collagen
HSP90‐α: heat shock protein 90 ‐ alpha
Il‐: interleukin – family, for example, 1 alpha, 2, 6
MMP‐2: matrix metalloproteinase ‐ 2
NGF‐β: nerve growth factor ‐ β
PAI‐1: plasminogen activator inhibitor ‐ 1
PO: peroxidase
TNF‐α: tumour necrosis factor‐alpha
TSLP: thymic stromal lymphopoietin
VEGF‐C: vascular endothelial growth factor ‐ C

## 1 INTRODUCTION

Skin is a multifunctional organ that is responsive to the external environment and the internal systemic condition of the individual.^1^ There is mounting evidence that the chemistry of the skin's surface can be correlated with underlying conditions and potentially with systemic changes.^2^, ^3^ Sampling the skin and wound surface for microbiological analysis is standard practise. A non‐invasive sampling technique for biomarker analysis is anticipated to be of interest for diagnosing and monitoring both skin pathology and systemic conditions.

Biomarkers are objectively quantifiable and measurable characteristics resulting from biological processes.^4^ Examples of skin biomarkers include testing of local (e.g., a swab to detect skin microbiota) or systemic status (e.g., biopsy to determine the level of myxovirus resistance protein A in cutaneous lupus).^5^, ^6^ Invasive tests, such as biopsy, remain a gold standard for comprehensive, full‐thickness analysis. However, a biopsy requires a skilled practitioner and has associated complications.

A broad definition of blotting is the transfer of biological substances from one medium to another.^7^ Blotting the skin to harvest material for surface chemistry analysis involves a nitrocellulose membrane applied to intact or broken skin for a period of 10 s to 10 min.^8^, ^9^ The blotting material is processed to identify the presence of biomarkers.

There has been no synthesis of the literature with respect to either skin or wound blotting. The primary objective of this study was to conduct a systematic review with narrative synthesis on blotting's applications for diagnosis and prognosis purposes. The secondary objectives were to present the typical blotting method with significance, variations, validity, and reliability to facilitate replication and guide future research. These results are anticipated to be helpful for wound care specialists, dermatologists and plastic surgeons working in clinical research.

### 1.1 Blotting related to skin physiology

**FIGURE 1:**
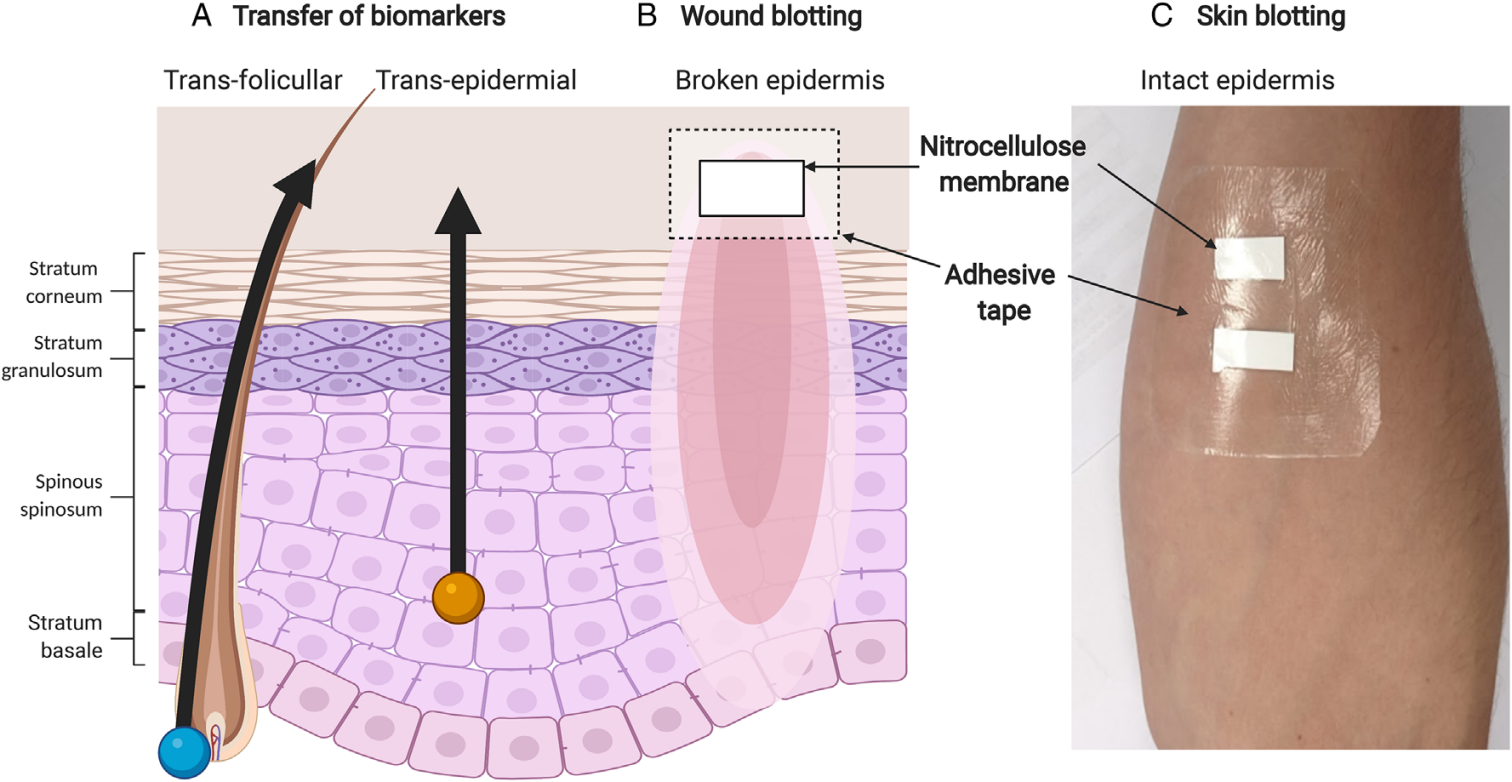
A diagram of microscopic skin physiology related to wound and skin blotting, showing the routes of molecules through the skin barrier.(A) Transfer of biomarkers. (B) Wound blotting. (C) Skin blotting [Color figure can be viewed at wileyonlinelibrary.com]

The skin is an effective barrier against irritants, pathogens and trans‐epidermal water loss due to the presence of sebum, intercellular lipids, and keratinocyte tight junctions.^10^ Under normal circumstances, only molecules <500 Daltons may penetrate the dermis.^11^ Minematsu et al. demonstrated that large water‐soluble molecules permeate in or out when the skin is over‐hydrated.^9^ Molecules within the deep dermis and subcutaneous tissue leak via the trans‐follicular route, whereas those in the more superficial layer of the dermis and epidermis permeate via the trans‐epidermal route. Minematsu et al. built on this concept proposed by Tanaka et al. to develop two blotting techniques.^12^ In the case of skin wounds, a fluid‐rich sub‐surface replete with cellular agents, messengers and pathogens is exposed.^13^ Both techniques use nitrocellulose membranes, commonly used to fix proteins in Western blotting. These attract polar molecules and absorb proteins.^9^ (see Figure 1). The commonly used steps are reported in Section 3.2.

## 2 MATERIALS AND METHODS

Investigators designed a protocol for a systematic review through the Centre for Open Science Framework before conducting data extraction – see https://doi.org/10.17605/osf.io/bynxq. This review complies with the PRISMA guidelines.^14^

### 2.1 Search strategy

Following PRISMA‐S guidance, a research librarian (CH) designed a systematic search strategy with investigators (see Appendix A).^15^ Searches were conducted on the Ovid Medline, Embase, and Google Scholar databases on 9 July 2021. Citation tracking was used for known active authors. Forward and backward tracking identified key papers that the PubMed and Google Scholar algorithms assessed as similar. Citations related to skin and wound surface chemistry sampling were included for background information. Search results were aggregated in EndNote software, deduplicated and shared with team members for screening.^16^ Through peer review and further citation searching, investigators found one eligible study and updated the synthesis to include this.^17^ There were no other deviations from the protocol.

### 2.2 Screening, data extraction and statistics

Investigators paired up to independently screen abstracts and full‐texts according to eligibility criteria – then extract data using a template (see Appendix B). In the case of unresolved disagreement, pairs reached a consensus through discussion. Descriptive statistics show population distributions with percentage frequencies, measures of central tendency and spread. No statistical testing was performed.

### 2.3 Eligibility criteria

Including:

- Publications in peer‐reviewed journals.
- Using skin or wound blotting defined by Minematsu et al.^8^, ^9^
- Participant species are animals or humans.
- Articles in any language.

Excluding: Conference abstracts, review papers.

### 2.4 Study quality

Using McMaster's Critical Review Form for Quantitative Studies, investigators independently assessed quality in pairs. This instrument determines the methodological quality of studies.^18^ Where there was disagreement, pairs reached a consensus by discussion. The assessment covers the domains of purpose, literature, design, outcome, intervention, results, dropouts, and conclusions. Each study was assigned an overall score out of 15. Based on the previous reviews, 7–10 was moderate quality and >10 was good quality.^19^

### 2.5 Reporting of biomarkers

Organic compound types can categorise biomarkers: proteins, carbohydrates, lipids, and nucleic acids. They are further sub‐categorised if there is a known association with a ‘pro’ or ‘anti’ effect in a recognised stage of wound healing. During processing, blots are stained or labelled to highlight biomarkers. Conventional histochemical dyes, such as Alcian blue, identify mucopolysaccharides, while immunostains recognise proteins such as tumour necrosis factor (TNF). Visualisation is facilitated by traditional light microscopy, chemical luminesce, or immunostaining. A computer can count and calculate the relative number of stained/labelled pixels. The reported results determine whether a biomarker is present, the spatial distribution of that biomarker on the blot, the measured area of the blot, and the quantity of the biomarker using immunoreactivity intensity.

### 2.6 Data synthesis

Some studies had two arms, a preliminary animal model and a human validation test. Both arms are reported in the text and tables. A summary of the skin and wound blotting steps is first presented, highlighting technique variations, validity, and reliability. This is followed by the role of blotting in associated skin physiology and disease, which has been categorised into skin or wound blotting.

## 3 RESULTS

**FIGURE 2:**
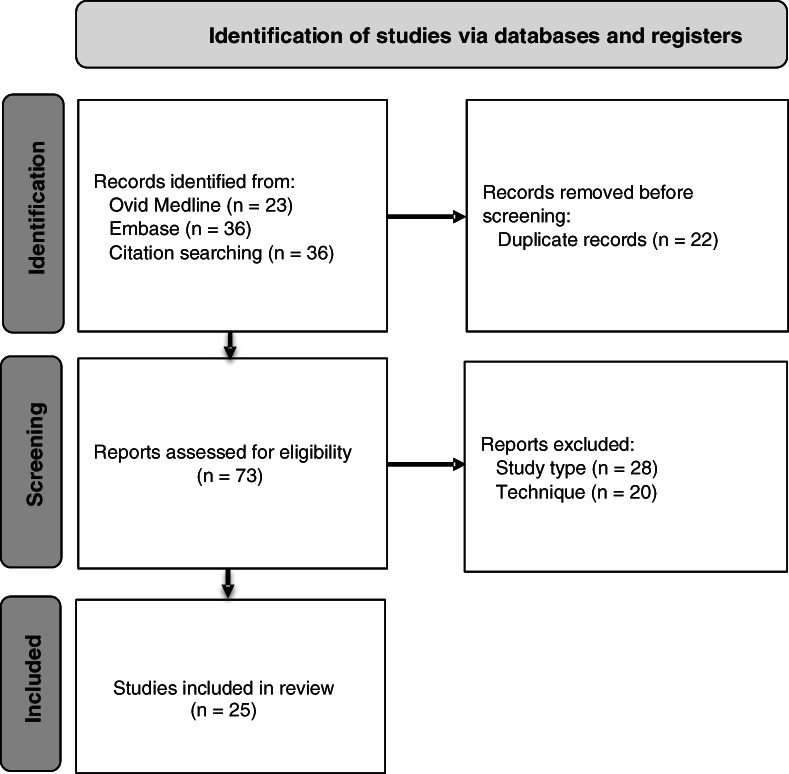
A PRISMA flow diagram showing the search, screening, exclusion and inclusion of studies^14^

Seventy‐three citations were screened. There were 25 studies categorised by skin (*n* = 14) or wound blotting (*n* = 11), undertaken in Japan (*n* = 22), Australia (*n* = 1), Indonesia (*n* = 1), and Taiwan (*n* = 1) between 2013 and 2021 (see Figure 2). Skin blotting was conducted on 101 animals and 1194 humans. Wound blotting was conducted on 75 animals and 352 humans (see Table 1).

**TABLE 1:** A summary of included studies

First author (year), Country	Skin or wound	Objectives	Study design	Population (*n*)	Age (years)Sex: [Male: Female]	Results	Quality
Minematsu (2013), Japan^8^	W	Assess the feasibility of blotting in a mouse model to detect levels and distribution associated with healing.	E	M (*n* = 5)	8 week old [5:0]	‐Blotting and immunostaining were valid for soluble proteins only ‐A non‐significant TNF distribution around the wound ‘edge’ appeared to be associated with delayed healing compared to the wound ‘bed’ ‐Blotting is the first technique to biochemically assess wounds in the epithelialisation phase	+
		Validate blotting in necrotic, full‐thickness pressure ulcers in relation to healing	RO	H (*n* = 2)	82 (median) [1:1]		
Minematsu (2014), Japan^9^	S	To assess the feasibility of blotting intact skin	E	M (*n* = 65)	7 week old [65:0]	‐Blotted values and histological analysis of F‐DEX and TNF indicated that deeper soluble proteins move via the trans‐follicular route and superficial molecules via the trans‐epidermal routes. ‐In humans, follicular TNF‐α values were significantly associated with BMI and consistently elevated in subjects with BMI greater than 30. ‐Immunohistochemistry of blotted proteins can be used to analyse secretion quantitatively in tissue sections	++
		To validate blotting biomarker TNF‐α levels in relation to route of passage through the skin and in relation to BMI amongst healthy volunteers.	CS	H (*n* = 59)	49 (median 49) [39:20]		
Kitamura (2015), Japan^20^	W	Predict 1‐week liquefaction of necrotic tissue through detection and distribution analysis of target proteins in pressure ulcers	RC	H (*n* = 12)	72 (mean) [6:6]	‐Statistically significant association between PO activity and 1‐week liquefaction of necrotic tissue, and between the heterogeneous distribution of PO and non‐liquefaction compared to homogenous and speckled distributions	++
Ogai **(**2015), *Japan* ^21^	S	Evaluate the use of total protein count to normalise individual variation in skin blotting results of TNF‐α between obese vs non‐obese.	E	H (*n* = 24)	Non‐obese: 43.7 (mean) Obese: 36 (mean) [24:0]	‐Blotted TNF‐α without normalisation did not show a statistically significant difference between healthy and obese skin (*p* = 0.69). After normalisation, the difference became statistically significant (*p* = 0.006), suggesting that normalisation may remove the variation between individuals.	+
Koyano (2016), *Japan* ^22^	S	Exploration of the prevalence of ST and the pre‐morbid skin properties associated with STs	CS	H (*n* = 36)	ST: 90 (median) No ST: 90.5 (median) [10:26]	‐ST were associated with reduced COL‐4 and MMP‐2 and increased TNF‐α blotted biomarkers. No significant differences in fibronectin levels were identified	++
Ogai (2016), *Japan* ^23^	S	Quantify the level of TNF‐α on the skin using blotting (with normalisation) in relation to the measures of obesity	E	H (*n* = 59)	40 (median) [59:0]	‐Statistically significant positive correlations were found between the levels of blotting TNF‐α and measures of obesity: weight, body fat, waist circumference and visceral fat rating	++
Koyano (2017), Japan^24^	S	Identify skin properties, including blotting, to predict the development of ST amongst older patients	PC	H (*n* = 142)	ST: 86 (median) No ST: 87 (median) [44:98]	There was no statistically significant association between ST and COL‐4, MMP‐2 and TNF‐α was detected by blotting.	++
Nakagami (2017), Japan^25^	W	Predict 1‐week slough development of PU by the post‐debridement presence of biofilm	PC	H (*n* = 16)	68 (median) [0:16]	‐Changes in wound slough formation can likely be predicted clinically using a non‐invasive wound blotting method. ‐Identification of biofilm by blotting led to a statistically significant increase in the odds‐ratio of slough in 1‐week follow‐up	++
Tamai (2017), *Japan* ^26^	S	Evaluate the blotting of Alb on mice after repeated tape stripping, then human skin without tape stripping, correlating with TEWL for skin barrier function.	E	M (*n* = 4)	8‐week‐old [4:0]	‐Alb intensity was statistically significantly correlated with TEWL values in the dorsum skin of mice (*p* = 0.02) and forearm skin of humans *p* < 0.01).	+
			CS	H (*n* = 9)	28 (median) [NS]		
Kitamura (2018), Japan^27^	W	Evaluate wound blotting proteins and distribution for PO and ALP with underlying histopathology specimens to reveal the sources of enzyme activity	E	R (*n* = 20)	9 week old [20:0]	‐Wounds with a high level of PO activity on blotting produced a ‘ring’ signal pattern. This pattern presents as a candidate marker to detect inflammation where inflammation is not visible	+
				R (*n* = 20)	6 month old [20:0]		
Koyano (2018), Japan^28^	S	Compare protein secretion on the intact skin of extremities and verify the relationship between the marker proteins on abdominal skin and systemic factors using skin blotting	CS	H (*n* = 70)	87 (median) [25:45]	‐Bland–Altman plots demonstrated no significant difference between right and left secretion levels on the forearms and lower legs amongst the three blotted biomarkers. ‐Multiple regression analysis showed that age and antiplatelet use was positively associated with decreased COL‐4 and increased MMP‐2, respectively. ‐Collecting blotting samples from either the right or left skin of the arm of the forearm or lower legs would be sufficient	++
Rayner (2019), Australia^17^	S	Assess risk factors for ST in aged care residents, including skin blotting to assess target proteins	PC	H (*n* = 173)	88 (mean) [50:123]	‐Blotted biomarkers showed no significant association with skin tears	++
Higuchi **(**2019) Japan^29^	S	Evaluate the skin of newborns with TEWL and blotting for target proteins to determine objective measures of skin problems	E	H (*n* = 7)	Neonates [5:2]	‐IL‐6 and TNF‐α measured using skin blotting were higher in rash‐presenting skin sites than in non‐rash‐presenting skin sites in the newborns. ‐Alb values by blotting have limited accuracy as a relative measure of skin problems during the neonatal period	++
Kitamura **(**2019), Japan^30^	W	Compare blotting of PO in PU against the DESIGN tool (macroscopic assessment of PU) to detect inflammation. Validate PO blotting in rats with human PU.	CS	R (*n* = 20)	6 month old [20:0]	‐A 2‐min ‘chemiluminescent’ test demonstrated the distribution of PO on the blotting membrane ‐Blotting showed external validity when demonstrated on human PUs, and necrotic tissue did not influence the detection of inflammation	+
			CS	H (*n* = 21)	78 (median) [13:8]		
Kunimitsu (2019), Japan^31^	W	Investigate the relationship between bacterial bioburden and the presence of inflammation in PU (detected by thermography)	CS	H (*n* = 98)	70 (mean) [56:42]	‐Biofilm presence was detected indirectly by staining the blotted membrane for the presence of polysaccharides. ‐A high bacterial count and positive test indicating biofilm formation were significantly associated with wound inflammation	++
Mori (2019), Japan^32^	W	Investigate wound blotting for point‐of‐care biofilm detection to examine the proportion of biofilm removal after ultrasonic debridement in chronic wounds	CS	H (No BWCS *n* = 64) (BWCS *n* = 16)	No BWCS: 77 (median) BWCS: 80 (median) [30:18]	‐Ultrasonic debridement had a superior capability to remove biofilms on clinical wounds, as detected by wound blotting	++
		Examine the effectiveness of ‘biofilm‐based wound care system (BWCS) for healing chronic wounds and ultrasonic debridement to promote wound healing	RC				
Nakai (2019), Japan^33^	S	Assess blotting as a prognostic tool to predict PU progression from stage I to II	CS	H (*n* = 19)	84 (median) [10:9]	‐The combination of positive VEGF‐C and HSP90‐α negative could be associated with the prognosis of stage I pressure ulcer *p* = 0.061 (non‐statistically significant findings). ‐PAI‐1, IL‐1α, VEGF‐C, and HSP90‐α could be biomarkers to predict stage I PU prognosis	++
Arisandi (2020), Japan^34^	S	Explore the blotting of TNF‐α as a risk factor for recurrent PU development within 2 weeks after conservative management	P	H (*n* = 20)	88 (median) [6:14]	‐No significant difference in TNF‐α level was found between patients with recurrent PUs and non‐recurrent PUs in healed PU skin or normal skin (*p* = 0.963 and *p* = 0.246, respectively)	++
Kimura (2020), Japan^35^	S	Using an animal model for PU development to demonstrate that the secretion of the candidate marker proteins in pressure‐loaded mouse skin can be detected by skin blotting	E	M (*n* = 32)	9 week old [32:0]	‐PAI‐1 showed no significant differences at any time point. ‐IL‐1α, VEGF‐C, and HSP90‐intensities were significantly higher at longer pressure loading times	++
Koyanagi (2020), Japan^36^	W	Examine the effect of local management on the biofilm area of pressure ulcers with critical colonisation	PO	H (*n* = 34)	80 (median) [21:13]	‐Blotting was used to detect the presence of a biofilm after iodine treatment. ‐Local management with iodine ointment guided by wound blotting may reduce biofilms of pressure ulcers	++
Nakagami (2020), Japan^37^	W	Determine whether biofilm elimination by debridement affects wound area decrease in pressure ulcers, confirmed using wound blotting	RC	H (*n* = 9)	75 (median) [6:3]	‐Blotting was used to detect the presence of a biofilm after debridement of wounds. ‐Biofilm‐based wound care guided by wound blotting may assist in eliminating bacterial bioburden more effectively for wound area reduction	++
Tamai (2020), Japan^38^	S	To determine the relationship between skin ultrasound images and muscle damage in wheelchair basketball athletes, using skin‐blotting examinations of the ischial regions	CS	H (*n* = 12)	27 (median) [12:0]	‐Muscle‐type CK‐M level in the fat infiltration group was significantly higher than in a non‐fat infiltration after training. ‐IL‐6 level in the fat infiltration significantly higher than in the non‐fat infiltration group after rest. ‐The combination of ultrasonographic images and skin blotting using CK‐M and IL‐6 could detect early deep tissue damage in wheelchair athletes	++
Wu (2020), Taiwan^39^	W	To develop a rapid tool for diagnosing wound biofilm presence by Alcian blue staining	E	H (*n* = 15)	59 (median) [NS]	‐Biofilm detection by staining correlated well with the clinical, microbiological culture assessment of chronic wounds (83.9% consistency; 95.2% sensitivity, and 60% specificity)	+
Astrada **(**2021), Japan^40^	W	To confirm the concurrent validity of wound blotting against PAGE for biofilm visualisation. To confirm the usability of Alcian blue as a substitute for Ruthenium red in detecting biofilms	E	R (*n* = 10)	7 week old [10:0]	‐Ruthenium red and Alcian blue were statistically correlated with PAGE for biofilms. ‐Alcian blue staining demonstrated greater sensitivity than Ruthenium red staining. ‐Because the Alcian blue staining is more convenient than Ruthenium red, wound blotting with Alcian blue would be a promising tool to guide clinicians in delivering biofilm‐based wound management	+
			E	H (*n* = 17)	61.8 (mean) [4:5]		
Sari **(**2021), *Indonesia* ^41^	S	Prediction of skin itch by blotting for target biomarkers, along with other objective measurements of skin status	CS	H (Control *n* = 319) (Itch *n* = 245)	70 (mean) [222:342]	‐Detection of Alb and NGF‐β by blotting were associated with the presence of itch (*p* < 0.001). ‐Subcutaneous hydration was significantly associated with a lower intensity level of NGF‐β and TSLP (*p* = 0.005, 0.003, respectively). ‐Skin pH was significantly associated with lower Alb, NGF‐β, and TSLP (*p* = 0.048, 0.035, and <0.001, respectively). ‐Alb, NGF‐β, and TSLP could be candidates for measuring itchy skin amongst older adults with disrupted skin barrier function and local skin inflammation	++

Abbreviations: Population: H, human; M, mice; R, rat. Age: IQR, interquartile range; NS, not specified; SD, standard deviation. Study designs: CS, cross‐sectional; E, experimental; PC, prospective cohort; PO, prospective observational; RC, retrospective cohort; RO, retrospective observational. Quality: +, moderate; ++, good.

### 3.1 Quality assessment

The 25 included studies were assessed across nine domains.^18^ In their overall score, 18 studies were deemed good quality, and 7 studies were of moderate quality.

### 3.2 Blotting technique

The blotting technique can be divided into four interventions: (1) surface preparation, (2) blot preparation, (3) blot application and removal, and (4) analysis shown in Table 2. Technique variation and biomarkers studies are shown in Table 3.

**TABLE 2:** The commonly reported steps for blotting

Wound	Skin	Wound
1. Surface preparation		
Anatomical location	Upper and lower limbs^a^	Wound surface^a^
Skincare	Avoiding ointments and skincare^a^	Debridement^a^
2. Blot preparation		
Hydration	Blot pre‐wet with saline	Blot not pre‐wet
Blot material	Nitrocellulose membrane	Nitrocellulose membrane
Size	50–264 mm^2^	100–400 mm^2^
3. Blot application and removal		
Duration	5–10 min	10 s to 1 min
Adhesion	Adhesive tape or similar	None
4. Analysis		
Storage	4°C	4°C
Processing	Immunostaining	Histochemical dye or immunostaining.
Reporting	Quantification of level; topographic distribution; functional analysis	Quantification of level; topographic distribution; functional analysis

^a^ Variation on a per study basis.

**TABLE 3:** A summary of blotting studies with their arms to demonstrate the techniques used

Paper	Species	Wet (volume)	Duration	Size (mm^2^)	Analysis	Anatomy for blotting	Biomarkers
Skin blotting							
Minematsu (2014)^9^	M	2 μl	1,5,10 min	100	Immuno	Dorsum	F‐DEX, TNF‐α
	H	5 μl		50^a^	Immuno	Posterior thigh	TNF‐α
Ogai (2015)^21^	H	10 μl	10 min	100	Immuno	2 cm to the left of the umbilicus	TNF‐α, Total Protein
Koyano (2016)^22^	H	2 μl	10 min	50^a^	Immuno	Dorsal forearm	COL‐4, Fibronectin, MMP‐2, TNF‐α
Ogai (2016)^23^	H	50 μl	10 min	100	Immuno	Abdomen & thigh	TNF‐α, total protein
Tamai (2017)^26^	H	‘drop’		*NS*	Immuno	Forearm	Alb
	M	‘drop’	10 min	100	Immuno	Dorsum	Alb
Koyano (2017)^24^	H	20 μl	10 min	*NS*	Immuno	Posterior forearm	COL‐4, MMP‐2, TNF‐α
Koyano (2018)^28^	H	20 μl		264	Immuno	forearms | abdomen |lower legs	COL‐4, MMP‐2, TNF‐α
Rayner (2019)^17^	H	NS		*NS*	Immuno	Upper &and lower extremity	COL‐4, MMP‐2, TNF‐α
Higuchi (2019)^29^	H	1 drop	10 min	100	Immuno	ankles | wrists | forehead | buttocks | chest | neck	Alb, IL‐1α, IL‐6, TNF‐α
Nakai (2019)^33^	H	2 μl	*NS*	100	Immuno	spinal column | rib | iliac crest | greater trochanter | upper rear iliac spine | sacrum | medial condyle | malleolus | fifth metatarsal head	Alb, HSP90‐α, IL‐1α, PAI‐1, VEGF‐C
Tamai (2020)^38^	H	50 μl	10 min	NS	Immuno	Ischium	CK‐M, IL‐6
Arisandi (2020)^34^	H	Pre‐wet	10 min	100	Immuno	(sacrum | coccyx | trochanter | scapula) & normal skin^b^	TNF‐α
Sari (2021)^41^	H	50 μl	10 min	100	Immuno	left forearm &| right forearm	Alb, IL‐2, NGF‐β, TSLP
Kimura (2020)^35^	M	50 μl	10 min	100	Immuno	dorsum	IL‐1α, HSP90‐α, PAI‐1, VEGF‐C,
Wound blotting							
Minematsu (2013)^8^	M	None	1 min	100	Immuno	dorsum	ALP, COL‐4, PO, TNF‐α
Kitamura (2015)^20^	H	None	10 s	NS	Immuno	sacral	ALP, MMP‐2, PO, TNF‐α
Nakagami (2017)^25^	H	None	10 s	NS	Red	sacrum |coccyx | ischial tuberosity | others	Mucopolysaccharides
Kitamura (2018)^27^	R	None	10 s	NS	Immuno	dorsum	ALP, PO
Kitamura 2019^30^	R	None		NS	Lumi	Dorsum	PO
	H	None	10 s	NS	Lumi and TPS	Sacrum | coccyx | greater trochanter | lateral malleolus | heel | shin | head | back | chest | knee	PO, Total protein
Mori (2019)^32^	H	Pre‐wet	10 s	NS	Blue	sacrum | coccyx | greater trochanter | others	Mucopolysaccharides
Kunimitsu (2019)^31^	H	None	10 s	NS	Red | blue	sacrum | coccyx | greater trochanter | others	Polysaccharide
Nakagami (2020)^37^	H	Pre‐wet		NS	Red | blue	Sacrum | others	Mucopolysaccharides
Koyanagi 2020^36^	H	None	10 s	NS	Red	trunk | limb	Exopolysaccharides
Wu (2020)^39^	H	None	10 s	NS	Blue	leg | foot | thigh | shoulder | hand | hip | sacrum | inguinal region	Polysaccharides
Astrada (2021)^40^	H	Pre‐wet		NS	Red | blue	trochanter | coccyx | leg | others	Exopolysaccharides
	M	None	10 s	400	Red | blue	Dorsum	

Abbreviations: Pre‐wet, blot wet but volume not specified; Wet, the volume of saline. Species: H, human; M, mice; R, rats. Analysis: Immuno, immunostaining; Lumi, chemiluminescence – staining to allow fluorescence; TPS, total protein staining; Red, ruthenium red, a stain for direct visualisation of saccharides; Blue, Alcian blue, a stain for direct visualisation of saccharides; NS, not specified. |, logical ‘or’. &, logical ‘and’.

a Size of blot: circular.

b Anatomy: ‘normal’ skin defined as contralateral or 5 cm superior and unaffected by pathology.

#### 3.2.1 Significance and variations

Surface preparation: The anatomical location varied if a localised pathology necessitated blotting at its site of occurrence, for example, a pressure ulcer. In studies testing the skin in unlocalised pathology, the upper and lower limbs were frequently tested (8/14 or 57% of human studies). Koyano et al. verified no difference in the bilateral protein secretion on the intact skin of extremities for type IV collagen (COL‐4), matrix metalloproteinase–2 (MMP‐2), and tumour necrosis factor‐alpha (TNF‐α).^28^ The paper demonstrated that systemic (e.g., age) and local factors (e.g. environmental exposures) influenced the intensity levels of COL4 and MMP‐2 on the extremities while systemic factors influenced abdominal COL4 and MMP‐2 (but not TNF‐α). Three skin blotting studies report controlling for cleaning or topical skincare agents, but this was not formally compared. Koyano et al. disallowed ointments or bathing the day before sampling to avoid disturbing protein balance.^28^ Higuchi et al. allowed daily bathing but did not permit moisturisers.^29^ Sari et al. reported blotting at least 30 min after ablutions or 1 h after bathing, and participants were asked to avoid skincare product applications.^41^ There were 9/11 (82%) studies that described a protocol for cleansing wounds before blotting. This was performed with normal saline or chlorhexidine solutions, except for three studies that did not specify a cleansing agent.

##### Blot preparation

Hydration: Pre‐wetting the membrane was conducted in all skin blotting studies; however, the volumes and solutions used varied or were not specified. Wetting the blot membrane (and thus over‐hydration of the skin) facilitates the passage of soluble molecules through the skin barrier.^9^ 3/9 (33%) wound blotting studies in humans wetted the membrane before application.

Blot material: Nitrocellulose membranes soak up soluble polar molecules and absorb proteins.^9^ Such membranes were used in 24/25 (96%) studies, while Wu et al. used a positively charged nylon transfer membrane (Biodyne B Nylon Membrane, PALL). This nylon transfer membrane was selected for its blotting, detection, and binding characteristics for all negatively charged molecules, making it ideal for the adhesion of polysaccharides.^39^

**Size**: For human skin blotting, the membrane size varied from 50 to 264 mm^2^. For wound blotting, only two studies specifically reported the size of the blotting membrane used (100 and 400 mm^2^).^8^, ^40^ Minematsu et al. and Koyano et al. used circular‐shaped skin blots, with the former justifying this as minimising the effect of tape (adhesive) removal on human skin.^9^, ^22^ All other studies reported square or rectangular blot dimensions or did not specify shape.

##### Blot application and removal

Duration: Most human skin blotting studies (12/13; 92%) applied the blot for 10 min versus 10 s for (10/11; 91%) wound studies. Minematsu et al. was the first study to describe skin blotting and blotted the backs of mice for 1, 5, and 10 min.^9^ The study found that immunostaining signals increased with increasing duration.

Adhesion: The method of using an adhesive backing to secure membrane adhesion to a surface was reported by 6/14 (43%) skin blotting studies. The use of adhesive backing ‘tape’ was most commonly used.^9^, ^21^, ^22^, ^28^, ^33^, ^41^ Adhesion was not reported by wound blotting studies. Hence, the surface influences the use of an adhesive agent. In skin blotting, the detachment of adhesive tape creates a risk of skin tears. In wound blotting, the wound size, edge and shape may not provide a surface for adhesion to be applied.

##### Processing

Storage: Following the application of nitrocellulose membranes to wounds, 13/25 (52%) studies described storing the membranes at 4°C after blotting but before analysis.

**FIGURE 3:**
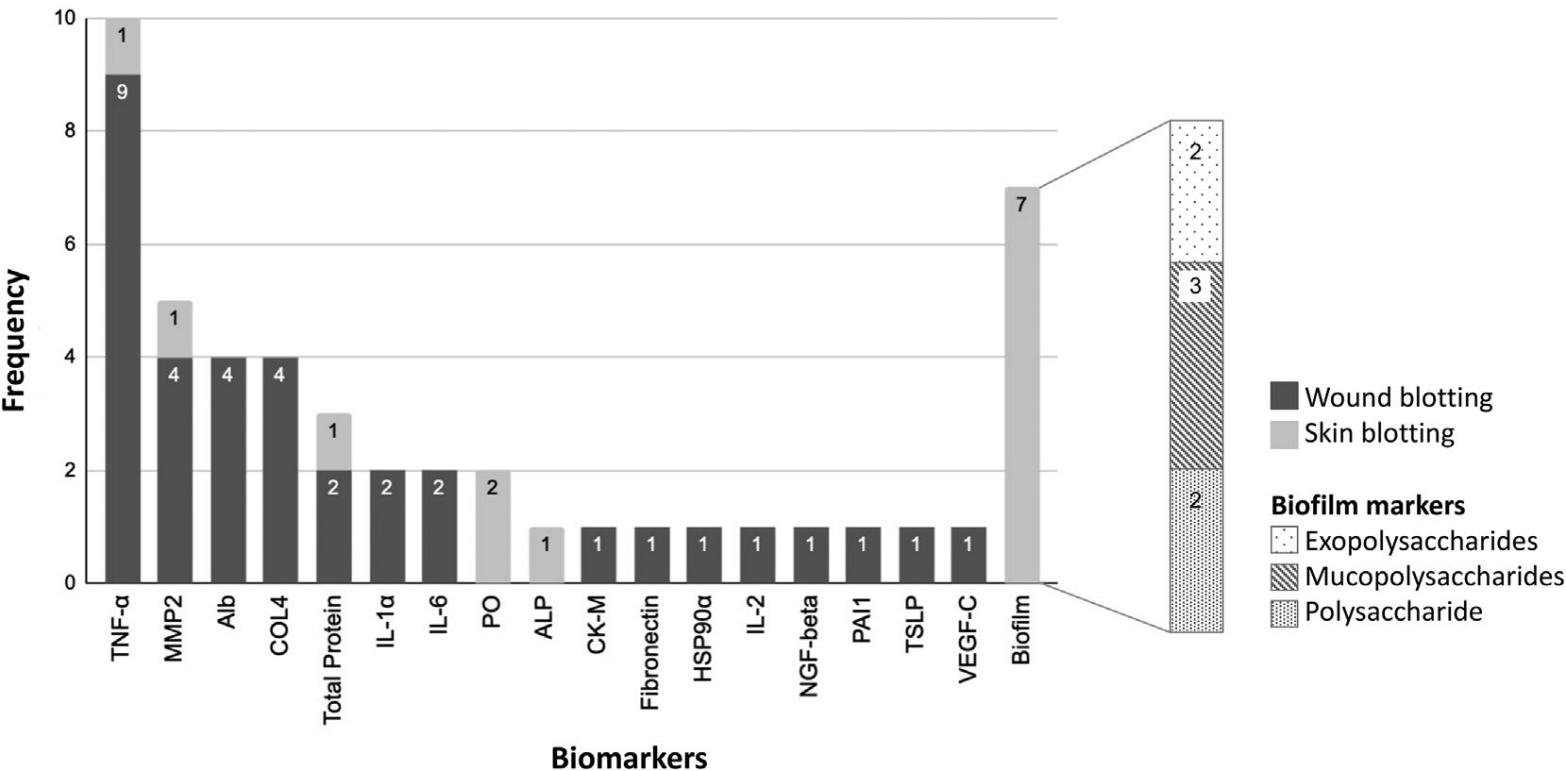
A stacked bar chart showing the distribution of reported biomarkers from blotting the skin and wounds of humans

Analysis: The blotting investigators used immunostaining or histochemical dye staining on the nitrocellulose membrane. The chemical agents and specific techniques varied per paper depending on the target biomarker. For Immunostaining, blocking solutions, single or double staining and chemiluminescent substrates were used to determine immunoreactivity. Seven studies used Ruthenium red or Alcian blue dyes for biofilm carbohydrate detection. Alcian blue dye was introduced by Wu et al. to replace Ruthenium red for a faster, cheaper, and more practical stain.^39^ Wu et al. and subsequently Astrada et al. confirmed the concurrent validity of wound blotting for biofilm visualisation and the usability of Alcian blue as a substitute for Ruthenium red.^39^, ^40^ Image processing software was used to evaluate densitometry. Twenty biomarkers were evaluated by skin (*n* = 14) and wound (*n* = 11) blotting studies (see Figure 3).

#### 3.2.2 Validity and reliability

##### Animal and in vitro models

Five studies used models to generate hypotheses before validating them with human samples. Minematsu et al. demonstrated that a mouse model was valid for skin and wound blotting for TNF‐α.^8^, ^9^ Tamai et al. validated the correlation of skin blotted albumin (Alb) with TEWL in rats before humans.^26^ Kitamura et al. used rat skin to demonstrate the validity of measuring peroxidase (PO) distribution as a marker of sub‐clinical inflammation in human wounds.^30^ Wu et al. used an in vitro model to detect biofilm on human specimens of *Pseudomonas aeruginosa* and *Staphylococcus aureus* before validating in vivo.^39^

##### Adjunct tests

Studies collected variables using clinical observations and measurement devices. These were used to confirm and validate clinical conclusions from blotting or compare blotting measurements.

For clinical observations, wound blot measurements were most commonly associated with pressure injury severity using the DESIGN‐R score.^25^, ^30^, ^31^, ^32^, ^33^, ^34^, ^36^ Koyano et al. and Tamai et al. used ultrasound, while Kunimitsu et al. used thermography to confirm tissue damage or clinical measurement.^22^, ^24^, ^31^, ^38^ Two studies used bacteria metres to associate blotted biofilm markers with a microbial count.^31^, ^32^ Four studies measured TEWL, although not all measured its direct association with blotted variables and three studies measured subcutaneous (SC) hydration and pH.^17^, ^22^, ^26^, ^29^, ^41^

Six studies used histological samples to improve criterion‐related validity from blotting. Minematsu et al. used tissue sections to confirm the passage of Fluorescein‐conjugated dextran (F‐DEX) and TNF via the trans‐follicular and trans‐epidermal routes.^9^ Kitamura et al. confirmed that the source of blotted PO was myeloperoxidase by observing extracellular deviation and secretion in tissue sections.^27^, ^30^ Kimura et al. used immunohistochemistry and haematoxylin and eosin‐stained tissue sections to reveal the tissue injury source of biomarkers in a rat model of pressure injury development.^35^ Astrada et al. demonstrated concurrent validity, identifying and quantifying biofilm on wounds compared with native polyacrylamide gel electrophoresis (PAGE) or ‘histological analysis by in vitro, in vivo and clinical investigations’.^40^

##### Accounting for individual differences

Koyano et al. demonstrated the reliability of blotting measurements between right and left, forearms and lower legs, for COL‐4, MMP‐2, and TNF‐α proteins, suggesting these are reliable sites for skin blotting.^28^ The individual skin variations seen with sex, age, race and body regions were accounted for by Ogai et al., who used total protein count curves to normalise the level of TNF‐α, allowing comparison between populations.^21^, ^42^

### 3.3 Skin blotting (intact skin)

#### 3.3.1 Skin tear prediction

Four papers explored the associations of COL‐4, MMP‐2, fibronectin and TNF‐α levels present in populations at risk of developing skin tears.^17^, ^22^, ^23^, ^24^ Koyano et al. and Ogai et al. found a significant association between raised TNF‐α levels in individuals over 65 years and obese males, respectively.^22^, ^23^ Koyano et al. later found no difference in TNF‐α levels nor significant changes of any blotted variables COL‐4 and MMP‐2 for skin tears using a multivariate model.^24^ Koyano et al. reported a significant decrease in COL‐4 and MMP‐2 (*p* = 0.042 and *p* = 0.028, respectively), while no association was found by Koyano et al.^22^, ^24^ Rayner et al. found no association between the blotted biomarkers tested and skin tears in a population of aged care residents in Australia.^17^

#### 3.3.2 Pressure injury prediction

Three studies evaluated the risk of pressure injuries (PI) using a permutation and combination of PAI‐1, IL‐1α, VEGF‐C, HSP90‐α and TNF.^33^, ^34^, ^35^

Development: Kimura et al. used a mouse model to predict the development of PI.^35^ Increased levels of IL‐1α, vascular endothelial growth factor – C (VEGF‐C), and heat‐shock protein 90 – alpha (HSP90‐α) were found to predict PI formation in a mouse model (p < 0.05). Tamai et al. 2020 tested pressure‐induced deep tissue injury amongst elite Japanese wheelchair basketball players using ultrasound and skin blotting instead of biopsy.^38^ A statistically significant association was found between deep tissue injury measured in post‐activity ultrasound images showing fat infiltration or low‐echoic/anechoic lesions with low levels of creatinine kinase – M (CK‐M) and high levels of IL‐6.

Progression: Nakai et al. found that the combination of VEGF‐C and HSP90‐α, detected over the nearby bony prominence from a PI, was a possible candidate biomarker to predict the progression of stage I to stage II PUs. However, this did not reach statistical significance (*p* = 0.061).^33^

Recurrence: Arisandi et al. found no significant difference between the TNF‐α levels of patients with recurrent PI and non‐recurrent PI in healed PI skin or normal skin (*p* = 0.963 and *p* = 0.246, respectively).^34^

#### 3.3.3 Newborn rash

Higuchi et al. measured Alb, IL‐1α, IL‐6 and TNF‐α levels in five‐day‐old baby skin with rash vs non‐rash skin. Raised IL‐6 and TNF‐α levels were significantly associated with rash‐presenting skin (*p* < 0.05). The detection sensitivity was 0.86, specificity 1, and an area under the curve of 0.92.^29^

#### 3.3.4 Skin barrier function

Skin barrier function, as measured by trans‐epidermal water loss (TEWL), was significantly correlated with the intensity level of Alb detected in the skin of older adults (*p* < 0.01) but not for 5‐day‐old newborns.^26^, ^29^ This suggests that Alb detected by blotting is a valid measure of skin barrier function. When evaluating subcutaneous (SC) hydration and pH, Sari et al. found that higher SC hydration was significantly associated with a lower intensity level of NGF‐β and TSLP (*p* = 0.005 and 0.003, respectively). The lower quantile of measured skin pH (indicating less disrupted barrier function) was significantly associated with lower levels of Alb, NGF‐β, and TSLP (*p* = 0.048, 0.035, and <0.001, respectively).^41^

#### 3.3.5 Pruritus

Sari et al. found that the signal levels of blotted Alb and NGF‐β were significantly higher in the cohort with itching than those without (*p* ≤ 0.001 and *p* < 0.001, respectively) and proposed that blotting for these two biomarkers may be candidates for the non‐invasive measurement of itch.^41^

#### 3.3.6 Obesity

Three studies evaluated skin fragility associated with obesity and surface TNF‐α level, suggesting this represents a possible test of skin's mechanical vulnerability in obese patients. Minematsu et al. found an association between TNF‐α levels in healthy male and female volunteers (*p* < 0.01).^9^ Ogai et al. conducted two studies using normalised TNF‐α values in healthy Japanese male skin. There was a significant association of measured TNF‐α levels with the severity of obesity measured by BMI, visceral fat rating, waist circumference, and body fat weight (*p* < 0.05).^21^, ^23^

### 3.4 Wound blotting (non‐intact skin)

Eleven studies used blotting for wounds. Seven studies examined biofilm detection, while the remaining four explored the detection of inflammatory biomarkers in wounds.

#### 3.4.1 Healing

Minematsu et al. reported the feasibility of blotting to detect TNF‐α, Alkaline Phosphatase (ALP) and COL‐4 in a mouse model followed by human pressure ulcers.^8^ TNF‐α distribution was categorised as ‘not present’, in the ‘edge’ of the wound, or the ‘bed’ of the wound. Categories suggested a potential association with healing but did not reach a significant conclusion.

Kitamura et al. evaluated progress towards healing measured by one‐week liquefaction of necrotic tissue (autolysis prior to granulation) with the distribution of PO, ALP, TNF‐α and MMP‐2.^20^ Higher PO activity levels and a non‐heterogeneous pattern were associated with liquefaction (*p* < 0.05).

#### 3.4.2 Biofilm

Biofilms were investigated in seven studies. Nakagami et al. prospectively took 70 blots from 16 patients with 23 pressure injuries and stained them for the presence of mucopolysaccharides, a biofilm component.^25^ The areas of wound and slough were recorded on the day of blotting and 1 week later. The odds ratio (OR) of the biofilm‐positive cases for an increased slough proportion, adjusted by the baseline DESIGN‐R total score, baseline percentage slough, and age, were 9.37 (*p* = 0.001), suggesting that the changes in wound slough formation can be predicted by blotting.

The relationship between bacterial count, biofilm presence and wound inflammation, based on thermography, was investigated by Kunimitsu et al.^31^ This cross‐sectional study on 273 samples from 98 patients with stage II (or deeper) pressure injuries revealed a non‐significant relationship between biofilm presence and wound inflammation (*p* = 0.076). However, bacterial count and biofilm presence, described as ‘bacterial bioburden’, was significantly associated with increased wound inflammation.

Mori et al. sought to combine blotting with a clinical intervention to produce a ‘biofilm‐based wound care system’ (BWCS) to promote wound healing.^32^ Chronic wounds, including pressure injuries, arterial ulcers, venous ulcers and diabetic ulcers, were blotted for biofilm presence. The biofilm‐positive wounds underwent low‐frequency ultrasonic debridement and subsequent blotting. The median biofilm removal proportion was 38.9% (interquartile range, 12.%–68%) for pressure injuries treated with standard care and 65.2% (41.1%–78.8%) for those treated with ultrasonic debridement (*p* = 0.009). The proportion of wound healing within 90 days was significantly higher in wounds treated with BWCS than in those treated with standard care (*p* = 0.001).

In a similar vein, Nakagami et al. explored the effect of biofilm elimination on the area of wound healing in pressure injuries 1‐week post sharp debridement.^37^ The percentage decrease in wound area was significantly higher in the biofilm‐eliminated group (median: 14.4%, interquartile range: 4.6%–20.1%) than in the biofilm‐remaining group (median: −14.5%, interquartile range: −25.3% to 9.6%; *p* = 0.04). Koyanagi et al. investigated the effect of six different topical treatments on the area of biofilm on 34 pressure injuries after 1 week.^36^ The use of iodine ointment was associated with a statistically significant reduction in biofilm area (*p* = 0.02).

In 2020, Wu et al. aimed to modify the wound blotting technique to establish a fast and straightforward procedure that is more clinically applicable.^39^ They employed Alcian blue rather than Ruthenium red staining, while the nitrocellulose membrane was replaced with a positively charged nylon transfer membrane. Biofilm presence in wounds could then be detected within a few minutes and staining results correlated well with microbiology culturing results (83.9% consistency, 95.2% sensitivity, and 60% specificity). Amongst the 18 cases with positive wound biofilm staining, 15 wounds (83.3%) were not healed at the 1‐month follow‐up visit (no statistical significance). Astrada et al. conducted in vivo and in vitro studies to confirm the concurrent validity of wound blotting for biofilm visualisation and the usability of Alcian blue as a substitute for Ruthenium red.^40^ The staining sensitivity of Ruthenium red was 88.9% and 100% for Alcian blue, and both had a good correlation with native PAGE analysis.

#### 3.4.3 Histopathological inflammation

Kitamura et al. used a rat model to look at the distribution of ALP and PO activity in wounds alongside histological specimens. The PO distribution on the wound edge but not on the wound bed (a ring signal) indicated an association with non‐visible inflammation. An association with ALP was not found.^27^ A finding confirmed in a more extensive 2019 study by the same author used a rat model validated in full‐thickness pressure injuries in humans. This study showed a significant association between DESIGN‐R signs of ‘inflammation/infection’ and ring signals (*p* = 0.016).^30^ Astrada et al. demonstrated that biofilm detection with Alcian blue provides evidence of the concurrent validity of wound blotting in identifying and quantifying biofilm on wounds compared with native PAGE or histological analysis by in vitro, in vivo, and clinical investigations.

## 4 DISCUSSION

This is the first synthesis of evidence on skin and wound blotting. Twenty‐five studies were systematically reviewed, demonstrating the applications for diagnosis and prognosis. Studies sampled protein and carbohydrate biomarkers in skin tears, pressure injuries, newborn rashes, pruritus, and biofilms, with physiological measurements of obesity, wound healing, and skin barrier function. Blotting is a valid and reproducible sample collection method for a wide range of biomarkers. It may have advantages over invasive tests for researchers and patients. Furthermore, it has the potential to be a bedside test. However, presenting a compendium of techniques across settings and physiology makes their reduction to a series of steps challenging. Blotting is not yet standardised, and future investigators should be mindful of adapting it to their needs.

Minematsu et al. cited the advantages of wound blotting as non‐invasiveness, repeatability without disturbing the wound, and sampling of the epithelialisation phase of healing.^8^ Similar non‐invasive skin tests are well documented.^42^ While a comparison to non‐invasive tests is beyond the scope of this study, the techniques identified during the literature search are summarised in Table 4. Further study is justified to compare techniques.

**TABLE 4:** A selective summary of biophysical tests for sampling the skin's surface chemistry

Technique (references)	Description	Application: example
FibroTx TAP (Schaap 2021)^43^	Transdermal Analysis Patch (TAP) is a proprietary nitrocellulose membrane primed with an array of antigens to detect proteins via subsequent immunostaining	Biomarkers in inflammatory skin disorders
Lavage of soluble biomarkers (Portugal‐Cohen 2013)^44^	A well is attached on the skin surface using an adhesive pad, filled with an extraction buffer for 30 min incubation of solubilised biomarkers, quantified using enzyme‐linked immunosorbent assay (ELISA)	Biomarkers in atopic dermatoses
Smart Sticker™ (Ferris 2018)^45^	A proprietary adhesive patch applied to a suspicious skin lesion, analysed for gene expression associated with malignant melanoma	Melanoma Gene Assay
Tape Stripping (He 2021)^46^	Adhesive tape (proprietary d‐Squame® tape discs or other) is applied to strip away layers to the depth of the upper granular layer of the epidermis	Biomarkers in psoriasis
DIUTHAME™ blotting (Kumata 2020)^47^	A proprietary Desorption Ionisation Using Through Hole Alumina Membrane (DIUTHAME™) applied to an organic surface to absorb biomarkers for imaging mass spectrometry	Imaging organic surfaces

### 4.1 Standardisation

Variations of the blotting method exist for skin and wound blotting, different pathologies and target biomarkers without apparent clinical reasoning. For example, the blot application duration was 10 min in 92% of skin studies versus 10 s in 91% of wound studies. Biomarkers in wound exudate pass to a blot membrane faster than through an intact epithelium.^8^, ^48^

The lack of blotting standardisation confers the advantage of the constant evolution of the method with the disadvantage of barriers to replicability. For example, Wu et al. tested and found nylon membranes superior to the commonly used nitrocellulose in biofilm sampling but reported the incomplete description of a cationic solution for blocking and washing impeded replicability.^39^

Even when the technique remained consistent, for example, the test–retest reliability of TNF‐α, COL‐4, and MMP‐2 associated with skin tear prediction was questionable. Koyano et al. found contradictory results in repeated testing in healthy volunteers, while Rayner et al. found no significant association in an older patient population, despite using the same method.^17^, ^22^, ^24^ Rayner controlled for the sampler, time of day, temperature and humidity, so population age/photoaging effects may account for some differences.^48^, ^49^ Standardisation of the method for specific populations could make findings more reliable.

Further variation between intact and broken epidermis can be seen in the solution to ‘wet’ the membrane and skin preparation, which varied across all studies without apparent reason. Additionally, the repeated detachment of adhesive tapes creates a possible risk of tearing in elderly skin.^28^ Our review highlights that more than one standardised protocol will exist to account for different clinical pictures and better clarify how the technique can affect results.

### 4.2 Skin blotting

TNF‐α was the most commonly investigated biomarker for the skin's fragility and inflammation amongst obese patients.^21^, ^23^ Biomarkers have been associated with conceptual models of pressure injury development. However, further studies are warranted to investigate the role of the candidate markers IL‐1α, VEGF‐C, HSP90‐α, VEGF‐C and CK‐M.^35^, ^38^, ^50^ The pathophysiological signatures of neonatal skin are still being elucidated.^51^ While further studies to investigate the predictive role of IL‐6 and TNF‐α levels are warranted, the non‐invasiveness of blotting is potentially of more benefit amongst neonates, in whom biopsy is ethically problematic and upsetting for patients and parents.^29^ Skin barrier failure is implicated in the aetiology of dermatitis and pruritus.^52^, ^53^ Its level of function is measurable with specific tools, e.g. TEWL, SC hydration, pH. Blotted Alb, NGF‐β, and TSLP may provide suitable alternatives, but further studies are needed to validate the measurements.^26^, ^41^

### 4.3 Wound blotting

Chronic wound beds with mature bacterial biofilms may contain proteins, glycoproteins, lipids, wound cell components, and DNA in an exopolymeric matrix (EPM).^54^ Specific DNA components of the EPM, including extracellular and neutrophil trapped bacterial DNA, can be distinguished from the components above with a biofilm membrane wound blot using commercially available nylon and cationic nitrocellulose membranes. These membranes were originally developed and used by molecular biology laboratory studies to do Southern blots to detect specific DNA fragments (or Northern blots to detect RNA and Western blots to detect protein).^55^ This adaptation of the technique has allowed EPM detection in addition to the exopolysaccharides, mucopolysaccharides, and polysaccharide biofilm molecules detected by the cationic dyes (Ruthenium red and Alcian blue).

Healing progress has been investigated during the epithelialisation phase of repair, but healing prognosis is not feasible on biomarkers alone.^8^, ^20^ The skin microbiota has an interplay with healing demonstrated through wound biofilms.^56^ For this purpose, blotting may be a candidate for a point‐of‐care bedside test. Mori et al. and Nakagami et al. demonstrated that blotting could detect biofilms at the bedside, while Kitamura et al. demonstrated that a blot and chemical luminescence at the bedside could non‐invasively show subclinical inflammation in PIs.^30^, ^32^, ^37^ Wu et al. refined the latter process to a 2‐min bedside test using Alcian blue staining nylon transfer membranes with biofilm components.^39^

### 4.4 Limitations of the method

McMaster's Quality Assessment Tool was selected for its standardised evaluation of method, given the heterogeneity of study designs and outcomes. The numeric thresholds for grading study quality as ‘moderate’ or ‘good’ were not adjusted when accounting for ‘not applicable’ results in assessed domains, which may have led to underestimating study quality. The grey literature and university theses databases were not searched, potentially missing contemporary blotting studies. Citation tracking during the search necessitated manual referencing, making search reproduction challenging. Meta‐analysis requires a narrow, measurable research question that was not possible in this review, given the breadth of the topic.

### 4.5 Limitations of included studies

Studies had a low level of evidence – the highest being level III.^57^ Only seven were prospective, while the remainder were retrospective, cross‐sectional, and experimental studies. Although associations are found, these are often novel and necessitate further testing. Additionally, studies had small sample sizes from single‐centres, predominantly in single countries and older age groups. Clinicians should use caution when applying the findings from these studies to other populations and care settings.

### 4.6 Implications of results for practice, policy, and future research

Further studies are needed to optimise the steps and variables in the blotting technique. The University of Tokyo research team, which authored 23/25 peer‐reviewed blotting studies, have routinely collected blotting samples since 2012.^37^ We recommend collaboration on future studies to expand to other research groups. While the translation of the technique to clinical settings presents logistical challenges, blotting has the advantage of being non‐proprietary and using commonly available resources. Future research topics might focus on sampling surface chemistry in pathologies such as scars, detecting lipid biomarkers, and using mass spectrometry for detection.

## 5 CONCLUSION

Blotting is a versatile, non‐invasive test of the skin and wound surface chemistry, which is valid and reproducible. This narrative synthesis systematically reviews its utility for diagnosing and making a prognosis in pre‐disease, pathological and physiological states. Skin blotting biomarkers may predict skin tears, pressure injuries, newborn skin problems, pruritis, and evaluating skin barrier function and fragility associated with obesity. Wound blotting has been used for predicting healing, biofilm presence and non‐visible inflammation. The steps for blotting are surface preparation, blot preparation, blot application and removal and analysis. Clinicians should be mindful that the blotting techniques have not been standardised across all settings. Further studies are needed to assess the effect of variation in technique to standardise the method, detect novel biomarkers, and appraise the technique against non‐invasive surface chemistry tests.

## FUNDING INFORMATION

Guy H. M. Stanley is the Cynthia Banham Research Fellow (Ian Potter Foundation; 2021) and received the Australian Government Research Training Programme Fees Offset Scholarship for a higher degree (University of Western Australia; 2022) towards funding this study. Mark Fear is supported by the Stan Perron Charitable Foundation, Perth Children's Hospital Foundation and Fiona Wood Foundation.

## CONFLICTS OF INTEREST

The authors have no conflicts of interest to declare.

## Data Availability

Data available on request from the authors ‐ The data that support the findings of this study are available from the corresponding author upon reasonable request.

## APPENDIX A SEARCH STRATEGY IN MEDLINE AND EMBASE

Ovid Medline ALL 1946 to 07 July 2021

(skin blot? Or skin blotting or wound blot? Or wound blotting).mp. 23

Embase 1974 to 07 July 2021 (skin blot? Or skin blotting or wound blot? Or wound blotting).mp.35

The searches were run on 09 July 2021. No limits or filters were applied to the search

When duplicates were removed, 22 unique citations were included in the initial review set.

### A.1. Citation tracking

As this non‐standardised technique, citation tracking was done for key authors. Forward and backward tracking was done (to find papers that cited key papers by known authors and papers that PubMed and Google Scholar judged to be similar to key papers according to their algorithms). References cited by key authors were also checked to identify citations not otherwise found.

#### A.1.1. Summary

Total citations provided to the team = 95.

Total unique citations found from a search in Medline and Embase = 59.

Additional citations found from citation tracking = 36.

Total citations provided for screening = 73.

## APPENDIX B DATA EXTRACTION TEMPLATE

**1. Publication details**

- Author names
- Publication year
- Study title
- Study type
- Objective/Aim
- Journal

**2. Wound or skin blotting?**

**3. Participants**

- Number and description of study arms
- Study eligibility criteria
- Sample size (n)
- Species in the study arms

**4. Participant Demographics**

- Participant Setting
- Ethnicity
- Gender (% male)
- Age
- BMI
- Design R
- Other

**5. Sample**

- Control anatomical sample site
- Experimental anatomical sample site
- Patient skin prep <24 h before blotting

**6. Technique of skin blotting**

- Technique of the analysis
- Methodology
- Size of blot
- Biomarkers collected by blotting
- Other non‐blotting variables

**7. Conclusions/Findings**
**8. Limitations**
**9. Clinical Importance**

## References

[1] Lopez‐Ojeda W , Pandey A , Alhajj M , Oakley AM . Anatomy, Skin (Integument). StatPearls (StatPearls Publishing; 2020. 28723009

[2] Paliwal S , Hwang BH , Tsai KY , Mitragotri S . Diagnostic opportunities based on skin biomarkers. Eur J Pharm Sci. 2013;50:546‐556. 23159445 10.1016/j.ejps.2012.10.009

[3] Zouboulis CC , Makrantonaki E . Clinical and laboratory skin biomarkers of organ‐specific diseases. Mech Ageing Dev. 2019;177:144‐149. 30118721 10.1016/j.mad.2018.08.003

[4] Biomarkers Definitions Working Group . Biomarkers and surrogate endpoints: preferred definitions and conceptual framework. Clin Pharmacol Ther. 2001;69:89‐95. 11240971 10.1067/mcp.2001.113989

[5] Zhu JL , Black SM , Chong BF . Role of biomarkers in the diagnosis and prognosis of patients with cutaneous lupus erythematosus. Ann Transl Med. 2021;9:429. 33842650 10.21037/atm-20-5232 PMC8033322

[6] Thong B , Olsen NJ . Systemic lupus erythematosus diagnosis and management. Rheumatology. 2017;56:i3‐i13. 28013206 10.1093/rheumatology/kew401

[7] blotting, n . OED Online. Oxford English Dictionary; 2021.

[8] Minematsu T , Nakagami G , Yamamoto Y , et al. Wound blotting: a convenient biochemical assessment tool for protein components in exudate of chronic wounds. Wound Repair Regen. 2013;21:329‐334. 23438022 10.1111/wrr.12017

[9] Minematsu T , Horii M , Oe M , et al. Skin blotting: a noninvasive technique for evaluating physiological skin status. Adv Skin Wound Care. 2014;27:272‐279. 24836618 10.1097/01.ASW.0000448461.25542.36

[10] Brandner JM , Zorn‐Kruppa M , Yoshida T , Moll I , Beck LA , de Benedetto A . Epidermal tight junctions in health and disease. Tissue Barriers. 2015;3:e974451. 25838981 10.4161/21688370.2014.974451 PMC4372028

[11] Bos JD , Meinardi MM . The 500 Dalton rule for the skin penetration of chemical compounds and drugs. Exp Dermatol. 2000;9:165‐169. 10839713 10.1034/j.1600-0625.2000.009003165.x

[12] Tanaka M , Takeda H , Ui K , Uchinuma E , Shioya N . Convenient evaluation of wound healing process by analysis of exudate on wound surface. Prog Med. 1998;18:180‐181.

[13] Broughton G , Janis JE , Attinger CE . The basic science of wound healing. Plast Reconstr Surg. 2006;117:12S‐34S. 16799372 10.1097/01.prs.0000225430.42531.c2

[14] Page MJ , McKenzie JE , Bossuyt PM , et al. The PRISMA 2020 statement: an updated guideline for reporting systematic reviews. BMJ. 2021;372:n71. doi:10.1136/bmj.n71 33782057 PMC8005924

[15] PRISMA‐S Group , Rethlefsen ML , Kirtley S , et al. PRISMA‐S: an extension to the PRISMA statement for reporting literature searches in systematic reviews. Syst Rev. 2021;10:39. 33499930 10.1186/s13643-020-01542-z PMC7839230

[16] The EndNote team . Clarivate. EndNote. 2013.

[17] Rayner R , Carville K , Leslie G , Dhaliwal SS . A risk model for the prediction of skin tears in aged care residents: a prospective cohort study. Int Wound J. 2019;16:52‐63. 30175484 10.1111/iwj.12985 PMC7948554

[18] McMaster's University . McMasters Quality Assessment Tool for Quantitative Studies. *Effective Public Health Practice Project* ; 2018 https://merst.ca/ephpp/

[19] Ritchie L , Wright‐St Clair VA , Keogh J , Gray M . Community integration after traumatic brain injury: a systematic review of the clinical implications of measurement and service provision for older adults. Arch Phys Med Rehabil. 2014;95:163‐174. 24016401 10.1016/j.apmr.2013.08.237

[20] Kitamura AMHS , Yoshida M , Minematsu T , et al. Prediction of healing progress of pressure ulcers by distribution analysis of protein markers on necrotic tissue: a retrospective cohort study. Wound Rep Reg. 2015;23:772‐777. 10.1111/wrr.12316 25976913

[21] Ogai K , Matsumoto M , Minematsu T , et al. Development of an improved method for quantitative analysis of skin blotting: increasing reliability and applicability for skin assessment. Int J Cosmet Sci. 2015;37:425‐432. 25712407 10.1111/ics.12217

[22] Koyano Y , Nakagami G , Iizaka S , et al. Exploring the prevalence of skin tears and skin properties related to skin tears in elderly patients at a long‐term medical facility in Japan. Int Wound J. 2016;13:189‐197. 24674027 10.1111/iwj.12251 PMC7949576

[23] Ogai K , Matsumoto M , Aoki M , et al. Increased level of tumour necrosis factor‐alpha (TNF‐α) on the skin of Japanese obese males: measured by quantitative skin blotting. Int J Cosmet Sci. 2016;38:462‐469. 26865211 10.1111/ics.12312

[24] Koyano Y , Nakagami G , Iizaka S , Sugama J , Sanada H . Skin property can predict the development of skin tears among elderly patients: a prospective cohort study. Int Wound J. 2017;14:691‐697. 27758078 10.1111/iwj.12675 PMC7950065

[25] Nakagami G , Schultz G , Gibson DJ , et al. Biofilm detection by wound blotting can predict slough development in pressure ulcers: a prospective observational study. Wound Repair Regen. 2017;25:131‐138. 28019691 10.1111/wrr.12505

[26] Tamai N , Minematsu T , Tsunokuni S , et al. Detection of albumin using skin blotting as a measure of skin barrier function. J Nurs Sci Eng. 2017;4:116‐120.

[27] Kitamura A , Minematsu T , Nakagami G , Sanada H . Assessment of histopathology of wounds based on protein distribution detected by wound blotting. SAGE Open Med. 2018;6:2050312118812220. 30455949 10.1177/2050312118812220 PMC6236855

[28] Koyano Y , Nakagami G , Minematsu T , Sanada H . Reliability of the skin blotting method when used on the elderly. Int Wound J. 2018;15:807‐813. 29897658 10.1111/iwj.12931 PMC7950027

[29] Higuchi S , Yoshida S , Minematsu T , Ichinose T . Detection of inflammatory cytokines by skin blotting as an objective measure of neonatal skin problems. J Nurs Sci Eng. 2019;6:33‐40.

[30] Kitamura A , Minematsu T , Nakagami G , et al. Assessing subclinical inflammation by peroxidase detection in patients with pressure ulcers. J Wound Care. 2019;28:586‐591. 31513504 10.12968/jowc.2019.28.9.586

[31] Kunimitsu M , Nakagami G , Kitamura A , et al. The combination of high bacterial count and positive biofilm formation is associated with the inflammation of pressure ulcers. Chronic Wound Care Manag Res. 2019;6:1‐7.

[32] Mori Y , Nakagami G , Kitamura A , et al. Effectiveness of biofilm‐based wound care system on wound healing in chronic wounds. Wound Repair Regen. 2019;27:540‐547. 31145519 10.1111/wrr.12738

[33] Nakai A , Minematsu T , Tamai N , Sugama J , Urai T , Sanada H . Prediction of healing in category I pressure ulcers by skin blotting with plasminogen activator inhibitor 1, interleukin‐1alpha, vascular endothelial growth factor C, and heat shock protein 90alpha: a pilot study. J Tissue Viability. 2019;28:87‐93. 30799135 10.1016/j.jtv.2019.02.002

[34] Arisandi D , Ogai K , Urai T , et al. Development of recurrent pressure ulcers, risk factors in older patients: a prospective observational study. J Wound Care. 2020;29:S14‐s24. 10.12968/jowc.2020.29.Sup4.S14 32279614

[35] Kimura N , Nakagami G , Minematsu T , Sanada H . Non‐invasive detection of local tissue responses to predict pressure ulcer development in mouse models. J Tissue Viability. 2020;29:51‐57. 31757582 10.1016/j.jtv.2019.11.001

[36] Koyanagi H , Kitamura A , Nakagami G , Kashiwabara K , Sanada H , Sugama J . Local wound management factors related to biofilm reduction in the pressure ulcer: a prospective observational study. Jpn J Nurs Sci. 2020;18:e12394. 33269552 10.1111/jjns.12394

[37] Nakagami G , Schultz G , Kitamura A , et al. Rapid detection of biofilm by wound blotting following sharp debridement of chronic pressure ulcers predicts wound healing: a preliminary study. Int Wound J. 2020;17:191‐196. 31680469 10.1111/iwj.13256 PMC7948602

[38] Tamai N , Minematsu T , Maeda T , Yabunaka K , Sanada H . The relationship between skin ultrasound images and muscle damage using skin blotting in wheelchair basketball athletes. Spinal Cord. 2020;58:1022‐1029. 32203066 10.1038/s41393-020-0442-6

[39] Wu Y‐F , Lee TY , Liao WT , Chuan HH , Cheng NC , Cheng CM . Rapid detection of biofilm with modified alcian blue staining: in‐vitro protocol improvement and validation with clinical cases. Wound Repair Regen. 2020;28:834‐843. 32691440 10.1111/wrr.12845

[40] Astrada A , Nakagami G , Minematsu T , et al. Concurrent validity of biofilm detection by wound blotting on hard‐to‐heal wounds. J Wound Care. 2021;30:S4‐s13. 10.12968/jowc.2021.30.Sup4.S4 33856931

[41] Sari DW , Minematsu T , Yoshida M , et al. Validity of skin blot examination for albumin and nerve growth factor beta to detect itching of the skin in Indonesian older adults. J Tissue Viability. 2021;30:42‐50. 33248877 10.1016/j.jtv.2020.10.001

[42] Serup J , Jemec GBE , Grove GL . Handbook of Non‐Invasive Methods and the Skin. CRC Press; 2013. doi:10.3109/9781420003307

[43] Schaap MJ , Bruins FM , He X , et al. Skin surface protein detection by transdermal analysis patches in pediatric psoriasis. Skin Pharmacol Physiol. 2021;34:271‐280. 34015784 10.1159/000516110 PMC8491489

[44] Portugal‐Cohen M , Kohen R . Non‐invasive evaluation of skin cytokines secretion: an innovative complementary method for monitoring skin disorders. Methods. 2013;61:63‐68. 23063704 10.1016/j.ymeth.2012.10.002

[45] Ferris LK , Gerami P , Skelsey MK , et al. Real‐world performance and utility of a noninvasive gene expression assay to evaluate melanoma risk in pigmented lesions. Melanoma Res. 2018;28:478‐482. 30004988 10.1097/CMR.0000000000000478

[46] He H , Bissonnette R , Wu J , et al. Tape strips detect distinct immune and barrier profiles in atopic dermatitis and psoriasis. J Allergy Clin Immunol. 2021;147:199‐212. 32709423 10.1016/j.jaci.2020.05.048

[47] Kuwata K , Itou K , Kotani M , Ohmura T , Naito Y . DIUTHAME enables matrix‐free mass spectrometry imaging of frozen tissue sections. Rapid Commun Mass Spectrom. 2020;34:e8729. 31951673 10.1002/rcm.8729

[48] Minematsu T , Yamamoto Y , Nagase T , et al. Aging enhances maceration‐induced ultrastructural alteration of the epidermis and impairment of skin barrier function. J Dermatol Sci. 2011;62:160‐168. 21498052 10.1016/j.jdermsci.2011.03.005

[49] Ayer J , Griffiths CEM . Photoaging in Caucasians. Cutaneous Photoaging. Vol 1–30. Royal Society of Chemistry; 2019. doi:10.1039/9781788015981-00001

[50] Schwartz K , Henzel MK , Ann Richmond M , et al. Biomarkers for recurrent pressure injury risk in persons with spinal cord injury. J Spinal Cord Med. 2020;43:696‐703. 31490098 10.1080/10790268.2019.1645406 PMC7534297

[51] Visscher MO , Carr AN , Winget J , et al. Biomarkers of neonatal skin barrier adaptation reveal substantial differences compared to adult skin. Pediatr Res. 2021;89:1208‐1215. 32599611 10.1038/s41390-020-1035-y PMC8119241

[52] Rosso JD , Zeichner J , Alexis A , Cohen D , Berson D . Understanding the epidermal barrier in healthy and compromised skin: clinically relevant information for the dermatology practitioner: proceedings of an expert panel roundtable meeting. J Clin Aesthet Dermatol. 2016;9:S2‐S8. PMC5608132 28936279

[53] Lee C‐H , Yu H‐S . Biomarkers for itch and disease severity in atopic dermatitis. Curr Probl Dermatol. 2011;41:136‐148. 21576954 10.1159/000323307

[54] Campoccia D , Montanaro L , Arciola CR . Extracellular DNA (eDNA). A major ubiquitous element of the bacterial biofilm architecture. Int J Mol Sci. 2021;22:9100. 34445806 10.3390/ijms22169100 PMC8396552

[55] Schooling SR , Hubley A , Beveridge TJ . Interactions of DNA with biofilm‐derived membrane vesicles. J Bacteriol. 2009;191:4097‐4102. 19429627 10.1128/JB.00717-08 PMC2698485

[56] Tomic‐Canic M , Burgess JL , O'Neill KE , Strbo N , Pastar I . Skin microbiota and its interplay with wound healing. Am J Clin Dermatol. 2020;21:36‐43. 32914215 10.1007/s40257-020-00536-w PMC7584558

[57] Howick J , Chalmers I , Glasziou P , et al. Explanation of the 2011 Oxford Centre for Evidence‐Based Medicine (OCEBM) Levels of Evidence (Background Document). Oxford Centre for Evidence‐Based Medicine; 2011.

